# Ursodeoxycholic Acid Does Not Improve COVID-19 Outcome in Hospitalized Patients

**DOI:** 10.3390/v15081738

**Published:** 2023-08-14

**Authors:** Francesca Colapietro, Giovanni Angelotti, Chiara Masetti, Dana Shiffer, Nicola Pugliese, Stella De Nicola, Francesco Carella, Antonio Desai, Monica Ormas, Marta Calatroni, Paolo Omodei, Michele Ciccarelli, Stefano Aliberti, Francesco Reggiani, Michele Bartoletti, Maurizio Cecconi, Ana Lleo, Alessio Aghemo, Antonio Voza

**Affiliations:** 1 Department of Biomedical Sciences, Humanitas University, Pieve Emanuele, 20072 Milan, Italyantonio.desai@humanitas.it (A.D.); michele.bartoletti@humanitas.it (M.B.); alessio.aghemo@hunimed.eu (A.A.);; 2 Division of Internal Medicine and Hepatology, Department of Gastroenterology, Humanitas Research Hospital IRCCS, 20089 Milan, Italymonica.ormas@humanitas.it (M.O.); 3 Humanitas Artificial Intelligence Center, Humanitas Research Hospital IRCCS, 20089 Milan, Italy; 4 Emergency Department, Humanitas Research Hospital IRCCS, 20089 Milan, Italy; 5 Nephrology and Dialysis Unit, Humanitas Research Hospital IRCCS, 20089 Milan, Italy; 6 Department of Gastroenterology, Division of Gastroenterology and Digestive Endoscopy, Humanitas Research Hospital IRCCS, 20089 Milan, Italy; paolo.omodei@humanitas.it; 7 Division of Respiratory Medicine, Humanitas Research Hospital IRCCS, 20089 Milan, Italy; 8 Infectious Disease Unit, Humanitas Research Hospital IRCSS, 20089 Milan, Italy; 9 Department of Anaesthesia and Intensive Care, Humanitas University IRCCS, 20090 Milan, Italy

**Keywords:** UDCA, COVID-19, ACE2

## Abstract

Ursodeoxycholic acid (UDCA) was demonstrated to reduce susceptibility to SARS-CoV-2 infection in vitro and improve infection course in chronic liver diseases. However, real-life evidence is lacking. We analyzed the impact of UDCA on COVID-19 outcomes in patients hospitalized in a tertiary center. Between January 2020 and January 2023, among 3847 patients consecutively hospitalized for COVID19, 57 (=UDCA group) were taking UDCA. The UDCA and the control groups (*n* = 3790) did not differ concerning comorbidities including diabetes mellitus type 2 (15.8% vs. 12.8%) and neoplasia (12.3% vs. 9.4%). Liver diseases and vaccination rate were more common in the UDCA group (14.0% vs. 2.5% and 54.4% vs. 30.2%, respectively). Overall mortality and CPAP treatment were 22.8 % and 15.7% in the UDCA, and 21.3% and 25.9% in the control group. Mortality was similar (*p* = 0.243), whereas UDCA was associated with a lower rate of CPAP treatment (OR = 0.76, *p* < 0.05). Treatment with UDCA was not an independent predictor of survival in patients hospitalized for COVID-19.

## 1. Introduction

Despite the development of vaccines and antiviral agents, COVID-19 is still a global health problem [1]. 

Emerging SARS-CoV-2 variants represent a clinical challenge due to increased transmissibility and immune escape power and consequent drug resistance mechanisms [2]. Moreover, several reports suggest reduced response rates to vaccines in at-risk groups [3].

So far, antivirals (Remdesivir, Paxlovid, Molnupiravir) are considered crucial both for prophylaxis and for treatment, but several limitations have been identified for their use.

Firstly, data on their efficacy are heterogeneous; for example, discrepant results have been produced with Remdesivir (Gilead Sciences, Foster City, CA, USA), an RNA-dependent RNA polymerase (RdRp) inhibitor with low oral bioavailability. In a double blind, placebo-controlled multicentric study [4,5], no benefits were seen in terms of clinical improvement [6] or shorter time of discharge in hospitalized patients treated with this intravenous derivative of GS-441524 [7]. In a large cohort of patients receiving Remdesivir, no significant effect was observed in terms of mortality, length of hospital stay, or need for ventilation [8]. Nonetheless, due to the acceptable safety profile and the impact on disease progression shown in several trials, Remdesivir is the first agent for SARS-CoV-2 approved by FDA, with particular recommendation in the early course of the disease [9].

Secondly, small molecule drugs suffer from high costs and safety issues that may limit their global access and applicability worldwide. 

Paxlovid (Pfizer, New York, NY, USA), an inhibitor consisting in a combination of nirmatrelvir (PF-07321332) with low dose ritonavir, is mainly recommended in mild-to-moderate COVID-19 [10]; due to its easily accessibility and oral formulation, it can be administrated in outpatients. However, its price [11] might be a barrier to treatment in low-income countries; moreover, both nirmatrelvir and ritonavir have raised several safety issues, the former concerning drug resistance profiles [12], and the latter drug–drug interactions [13].

Similar constraints affect Molnupiravir (Merck, Rahway, NJ, USA), an orally available antiviral which is limited in efficacy by resistance-associated substitutions found in emerging SARS CoV-2 variants [14]. 

Approval of new drugs with demonstrated efficacy, enhanced oral bioavailability, wide global access, and low adverse events is needed. Ursodeoxycholic acid (UDCA), a naturally produced secondary bile acid largely used in cholestatic liver diseases, is a first-line treatment for primary biliary cholangitis (PBC) [15]. UDCA is also administered long-term in patients undergoing hematopoietic stem cell transplantation or those affected by bone marrow diseases to prevent veno-occlusive disease and cholangiopathy [16]. Recently, Brevini et al. [17] reported that UDCA can reduce susceptibility to SARS-CoV-2 infection via downregulation of ACE2 in vitro, in vivo, and in human lungs and livers perfused ex situ. In vivo, the same authors showed that UDCA treatment improved clinical outcomes after SARS-CoV-2 infection in their analysis of retrospective registry data. They confirmed these findings in an independent validation cohort of liver transplantation recipients. However, these clinical results were obtained only in two small cohort of patients with liver diseases receiving UDCA (*n* = 31 and *n* = 24). With the aim of assessing the real-life efficacy of UDCA in terms of reducing mortality and the need for continuous positive airway pressure (CPAP) in COVID-19 hospitalized patients, we analyzed all patients consecutively hospitalized with COVID-19 at our center between 2020 and 2023.

## 2. Material and Methods

We designed a retrospective, single-center, observational case–control study including all patients with SARS-CoV-2 infection admitted to Humanitas Research Hospital (Milan, Italy) from January 2020 to January 2023. All patients on UDCA treatment at therapeutic dosage for at least 3 months at hospital admission were defined as the UDCA group and compared to those not receiving UDCA (control group). Indications for UDCA included cholelithiasis, cholestatic liver diseases, and bone marrow diseases. 

Data were extracted from the electronic health record (EHR) via Structured Query Language (SQL). A total of 33 features were collected for each patient, including demographics (age, sex), comorbidities (arterial hypertension, AH; diabetes mellitus, DM; chronic kidney disease, CKD; concomitant neoplasia; liver disease; hearth disease; and chronic obstructive pulmonary disease, COPD), COVID-19 vaccinations, and biochemistry data such as liver function tests (LFTs) and platelet (PLT) count. 

The outcomes assessed in this study were the need for CPAP treatment, either at admission or during hospitalization, and in-hospital mortality.

Statistical analysis was performed using Python 3.9 via Scikit-Learn version 1.2.2 and scipy version 1.10.1. Modeling was performed via a class-weighted logistic regression to account for imbalances in the sample. 

Comorbidities were further processed by leveraging regular expressions from admission notes. Continuous features were processed via a quantile mapping to a gaussian distribution to respect the assumptions of normality required by the linear estimator. The model was then evaluated via 10-fold nested stratified cross-validation optimized for F1-score maximization. The choice of a linear estimator was supported by the need to maintain some interpretability of the classification, namely the possibility of evaluating pseudo-odds-ratios associated with covariates. For each outcome, a different model was trained and evaluated to assess the association with UDCA. Chi squared test was used to evaluate the potential impact of UDCA in the pre- and post-COVID-19 vaccination periods.

## 3. Results

### 3.1. Patients Characteristics

Between January 2020 and January 2022, 3847 patients were admitted and hospitalized in our center for SARS-CoV-2 infection. Of them, 57 patients (1.5%) were on chronic UDCA treatment at a therapeutic dosage (9.7 mg/kg daily). Indications for UDCA treatment were cholelithiasis (*n* = 18, 31.6%) and cholestatic diseases (*n* = 11, 19.3%); nine patients (15.8%) were affected by hematologic diseases including bone marrow diseases or stem cell transplantation. In Table 1, the demographic, biochemical, and clinical characteristics of UDCA group and control group (*n* = 3790) are shown and compared.

Patients in the UDCA group were slightly younger than patients in the control group (mean age 70.0, 58.0–82.0, vs. 74.0, 61.0–83.0, *p* < 0.001) with a higher prevalence of COPD (12.3% vs. 9.0%, OR 2.20 (1.39–2.73), *p* < 0.001), CKD (14.0% vs. 7.7%, OR 2.35 (2.10–2.06), *p* < 0.001), heart disease (26.3% vs. 19.7%, OR 2.43 (2.18–2.94), *p* < 0.001), and, as expected, chronic liver disease (14.0% vs. 2.5%, OR 5.38 (2.99–7.05), *p* < 0.001). It is worth noting that the distribution of diabetes mellitus and neoplasia was similar between the two groups. Patients in the UDCA group had a higher rate of COVID-19 vaccination compared to the control group (54.4% vs. 30.2%, OR 1.39 (95% CI 1.10–1.68), *p* < 0.001).

### 3.2. Outcomes

The mortality rate during hospitalization was 22.8% in the UDCA group and 21.3% in the control group (*p* value). COVID-19 vaccination was independently associated with lower mortality (OR 0.79, CI: 0.70–0.90, *p* < 0.01); thus, we divided the cohort in two groups, those hospitalized in the pre-vaccination period (from March 2020 to January 2021) and those hospitalized in the post-vaccination period (from February 2021 until the end of the study). While we observed a decrease in mortality from 26.7% to 21.4% in the UDCA group, and from 22.1% to 20.6% (*p* = 0.475) in the control group, we found no significant difference in the mortality rate between the pre-vaccination period (from March 2020 to January 2021) and the post-vaccination period (from February 2021 until the end of the study) in relation to UDCA exposure. COVID-19 vaccination was also associated with a reduced need for CPAP treatment (OR:0.58, CI: 0.49–0.63, *p* < 0.01). The use of CPAP treatment was also less common in the UDCA group (9/57, 15.8%) than in the control group (983/3790, 25.9%). 

**Figure 1:**
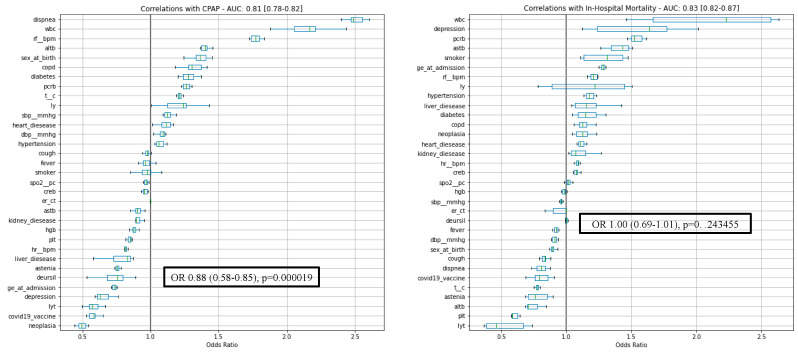
Correlations with CPAP treatment and in-hospital mortality. CPAP: continuous positive airway pressure.

After training, our model(s) found no significant association between mortality and exposure to UDCA (OR: 1.00 CI: 0.69–1.01, *p* = 0.243) despite the robust separating power of the model (AUC: 0.83 CI = 0.82–0.87, F1Pos: 0.59 [0.57–0.63]). On the other hand, UDCA treatment seemed to reduce the need for CPAP treatment (OR: 0.76 CI: 0.58–0.85, *p* << 0.05, AUC: 0.81 CI: 0.78,0.82, F1Pos: 0.58 CI: 0.55–0.59) (Figure 1).

## 4. Discussion

The development of safe, inexpensive, and effective treatments for SARS-CoV-2 is crucial for improving COVID-19 outcomes, particularly in high-risk groups such as immunosuppressed patients who may have a limited response to vaccination. Remdesivir is the first FDA-approved anti SARS-CoV-2 agent, but recent trials raise concerns about its efficacy in terms of reduction of mortality rate or length of hospitalization due to controversial results; moreover, its limited oral bioavailability decreases the accessibility of Remdesivir; indeed, patients need intravenous administration in the hospital setting. On the other hand, the effectiveness of monoclonal antibodies is reduced by viral variants, while universal use of antivirals is unfeasible due to elevated healthcare costs [18]. Given all these factors, small molecules may play a crucial role in treating COVID-19 due to oral administration, increased accessibility and affordability thanks to limited costs, and reduced side effects.

UDCA is an orally administered drug that is readily available, affordable, and has a favorable safety profile with minimal drug–drug interactions [19,20]. Next to its indication as the gold standard treatment in PBC, long-term, off-label use of UDCA is common in patients with lithiasis of the biliary tract or hematological diseases in several European countries, including Italy [21,22]. UDCA reduces susceptibility to SARS-CoV-2 infection by reducing ACE2 levels in lungs and nasal epithelium [17] via suppression of FXR activity. In an analysis of two registries involving patients with liver diseases, Brevini et al. found an association between UDCA treatment and reduced mortality, fewer ICU admissions, and shorter hospital stays. In contrast to this finding, Liu T and Wang JS very recently performed a survey in a pediatric population including 280 patients treated with UDCA for liver diseases, demonstrating that UDCA does not reduce susceptibility to SARS-CoV-2 infection [23]. On the other hand, John et al. [24] lately confirmed the positive impact of UDCA on clinical outcomes in COVID-19 in a retrospective cohort with limitation to patients with liver cirrhosis, with consequent selection bias. A second study limited to adult subjects with liver diseases enforced these results, but its survey design performed SARS COV-2 infection diagnosis biased based on patients’ self-report rather than experimental investigations [25].

Based on this, the main strength of our analysis was to not fully replicate the findings of these previous studies due to the inclusion of a larger cohort of hospitalized patients, with only a few individuals having an underlying liver disease. Specifically, we did not observe any impact of UDCA on in-hospital mortality. However, we did find a slight improvement in the COVID-19 course among patients treated with UDCA in terms of a lower need for CPAP treatment (OR 0.76). We intentionally did not analyze ICU admission or length of hospitalization as these surrogate endpoints might be biased by several confounding factors such as frailty, age of the patients, and ICU availability during the first wave of the COVID-19 pandemic. Additionally, the lower rate of CPAP treatment in patients with concurrent neoplastic diseases may reflect a less aggressive approach to treatment in individuals with severe comorbidities or may be influenced by clinical judgment.

Our study has certain limitations that should be considered. Firstly, the relatively small proportion of patients receiving UDCA (1.5% of the cohort) prevented us from using propensity score matching for our statistical analysis. Due to retrospective design of the study, information concerning the duration of UDCA treatment could not be retrieved for potential correlation with outcomes. Additionally, the widespread implementation of COVID-19 vaccination in our region starting from February 2021 had a significant impact on the outcomes. Despite these limitations, our findings indicate that UDCA at therapeutic dosage did not have a significant impact on in-hospital mortality in COVID-19 patients with and without liver disease, due to potential off-label use of UDCA in our country. This suggests that future studies on UDCA should probably focus on patients with less severe disease or, given the lack of significant drug–drug interactions (search on Liverpool Database, 8 August 2023 [26]), explore combinations with other approved regimens. 

## Figures and Tables

**Table 1:** Description of demographic and clinical features of UDCA-taking group, control group and total cohort.

	UDCA-Taking Group (*n* = 57)	Control Group (*n* = 3790)	Total Group (*n* = 3847)	OR (95% CI)	*p*-Value
**Age** (years) (mean, IQR)	70.0 (58.0–82.0)	74.0 (61.0–83.0)	74.0 (61.0–83.0)	0.87 (0.81–0.98)	0.000166
**Sex** (*n*, %)					
Male	31 (54.4%)	2296 (60.6%)	2327 (60.5%)	0.83 (0.70–0.98)	0.000393
Female	26 (45.6%)	1494 (39.4%)	1520 (39.5%)		
**Comorbidities** (*n*, %)					
Arterial Hypertension	10 (17.5%)	1020 (26.9%)	1030 (26.8%)	0.36 (0.24–0.43)	0.000000001
Diabetes Mellitus type 2	9 (15.8%)	520 (12.8%)	529 (13.8%)	0.95 (0.63–1.21)	0.707347
Chronic Obstructive Pulmonary Disease	7 (12.3%)	342 (9.0%)	349 (9.1%)	2.20 (1.39–2.73)	0.000048
Heart Disease	15 (26.3%)	746 (19.7%)	761 (19.8%)	2.43 (2.18–2.94)	0.00000006
Chronic Kidney Disease	8 (14.0%)	293 (7.7%)	301 (7.8%)	2.35 (2.10–2.06)	0.00000049
Liver disease	8 (14.0%)	96 (2.5%)	104 (2.7%)	5.23 (2.99–7.05)	0.000001
Neoplasia	7 (12.3%)	358 (9.4%)	365 (9.5%)	0.91 (0.62–1.20)	0.239422
**COVID-19 vaccination** (*n*,%)					
Vaccination	31 (54.4%)	1145 (30.2%)	1176 (30.6%)	1.39 (1.10–1.68)	0.000329
No vaccination	26 (45.6%)	2645 (69.8%)	2671 69.4%)		
**Biochemistry** (mean, range)					
AST (IU/L)	27.0 (20.75–52.75)	30.5 (22.0–44.38)			
	26.75 (15.50–50.25)	29.32 (18.19–51.5)			
ALT (IU/L)					
	9 (15.8%)	983 (25.9%)			
**Outcomes** (*n*,%)	13 (22.8%)	806 (21.3%)			
CPAP			992 (25.8%)	0.76 (0.58–0.85)	0.000019
In-hospital deaths			819(21.3%)	1.00 (0.69–1.01)	0.243455

AH: arterial hypertension; ALT: alanine aminotransferase; AST: aspartate aminotransferase; CKD: chronic kidney disease; CI: confidence interval; COPD: chronic obstructive pulmonary disease; CPAP: continuous positive airway pressure; DM type 2: diabetes mellitus type 2; ICU: intensive care unit; IQR: interquartile range; OR: odds ratio.

## Data Availability

The data that support the findings of this study are available from the corresponding author upon reasonable request.

## Abbreviations

UDCA: ursodeoxycholic acid; PBC: primary biliary cholangitis; CPAP: continuous positive airway pressure; HER: electronic health record; SQL: Structured Query Language; AH: arterial hypertension; DM: diabetes mellitus; CKD: chronic kidney disease; COPD: chronic obstructive pulmonary disease; LFT: liver function test; PLT: platelets; OR: odds ratio; CI: confidence interval; ACE2: angiotensin-converting enzyme 2; FXR: farnesoid X receptor; ICU: intensive care unit; IQR: interquartile range.
